# An evaluation of ICD-11 posttraumatic stress disorder criteria in two samples of adolescents and young adults exposed to mass shootings: factor analysis and comparisons to ICD-10 and DSM-IV

**DOI:** 10.1186/s12888-016-0849-y

**Published:** 2016-05-12

**Authors:** Henna Haravuori, Olli Kiviruusu, Laura Suomalainen, Mauri Marttunen

**Affiliations:** Department of Health, Mental Health Unit, National Institute for Health and Welfare, P.O. Box 30, FI-00271 Helsinki, Finland; Adolescent Psychiatry, University of Helsinki and Helsinki University Hospital, HUS, P.O. Box 590, FI-00029 Helsinki, Finland

**Keywords:** Posttraumatic stress disorder, PTSD, Mass shooting, ICD-11, ICD-10, DSM-IV, Adolescent, Young adult

## Abstract

**Background:**

The proposed posttraumatic stress disorder (PTSD) criteria for the International Classification of Diseases (ICD) 11th revision are simpler than the criteria in ICD-10, DSM-IV or DSM-5. The aim of this study was to evaluate the ICD-11 PTSD factor structure in samples of young people, and to compare PTSD prevalence rates and diagnostic agreement between the different diagnostic systems. Possible differences in clinical characteristics of the PTSD cases identified by ICD-11, ICD-10 and DSM-IV are explored.

**Methods:**

Two samples of adolescents and young adults were followed after exposure to similar mass shooting incidents in their schools. Semi-structured diagnostic interviews were performed to assess psychiatric diagnoses and PTSD symptom scores (*N =* 228, mean age 17.6 years). PTSD symptom item scores were used to compose diagnoses according to the different classification systems.

**Results:**

Confirmatory factor analyses indicated that the proposed ICD-11 PTSD symptoms represented two rather than three factors; re-experiencing and avoidance symptoms comprised one factor and hyperarousal symptoms the other factor. In the studied samples, the three-factor ICD-11 criteria identified 51 (22.4 %) PTSD cases, the two-factor ICD-11 identified 56 (24.6 %) cases and the DSM-IV identified 43 (18.9 %) cases, while the number of cases identified by ICD-10 was larger, being 85 (37.3 %) cases. Diagnostic agreement of the ICD-11 PTSD criteria with ICD-10 and DSM-IV was moderate, yet the diagnostic agreement turned to be good when an impairment criterion was imposed on ICD-10. Compared to ICD-11, ICD-10 identified cases with less severe trauma exposure and posttraumatic symptoms and DSM-IV identified cases with less severe trauma exposure.

**Conclusions:**

The findings suggest that the two-factor model of ICD-11 PTSD is preferable to the three-factor model. The proposed ICD-11 criteria are more restrictive compared to the ICD-10 criteria. There were some differences in the clinical characteristics of the PTSD cases identified by ICD-11, when compared to ICD-10 and DSM-IV.

## Background

The International Classification of Diseases is currently under revision for an 11th version by the World Health Organization (WHO). WHO has pursued clinical utility in the diagnostic criteria for mental disorders, with the intended aim of implementing simplicity and a limited set of symptoms [1]. The proposed ICD-11 Beta Draft criteria for posttraumatic stress disorder (PTSD) include exposure to a *threatening or horrific event or series of events* followed by symptoms from each of the three core elements: re-experiencing of the traumatic event(s) in the present day with emotions of fear or horror; avoidance of traumatic reminders; sense of a current threat manifested as hypervigilance and/or an exaggerated startle response; and having symptoms lasting for several weeks [2, 3]. A difference from the earlier version of ICD-10 is that functional impairment is now also required [2, 4]. Moreover, it has been proposed that ICD-11 would include a complex PTSD diagnosis with additional features of affect dysregulation, negative self-concept and interpersonal problems [5].

The proposed ICD-11 criteria imply that there would be three PTSD symptom factors (re-experiencing, avoidance and hyperarousal), although the latent structure of the symptoms has not been thoroughly studied [6]. Studies with DSM-5 PTSD symptoms indicate a very high correlation between re-experiencing and avoidance symptoms [7, 8]. Forbes et al. [6] studied the latent factor structure of the ICD-11 PTSD: comparisons were made between the three-factor model, where one out of two symptoms for each factor is required for the diagnosis; a two-factor model that combines re-experiencing and avoidance symptoms, where two out of four of these symptoms are required for the diagnosis; and finally a one-factor model. Confirmatory factor analysis (CFA) showed that the two-factor model had at least an equivalent fit with the original three-factor model, and being a more parsimonious model, is the preferred one [6]. Point prevalence rates were slightly higher with the two-factor solution. The authors suggested that the alternative two-factor model and diagnostic algorithm be used [6]. Tay et al. and Hansen et al. have performed CFAs on the ICD-11 three-factor model that showed a good model fit, although neither study analysed alternative factor models [9, 10].

Meanwhile, the Diagnostic and Statistical Manual of Mental Disorders, 4th Edition (DSM-IV) and the current 5th edition (DSM-5) [11, 12] are used in research and clinical practice simultaneously with ICD-10. The DSM-5 PTSD criteria have been criticized for being too complex with ambiguous four symptom clusters, making diagnostic assessment in clinical practice exhaustive [10, 13].

Changes in classification systems may have implications on the prevalence rate estimates of disorders, changes in clinical features such as comorbidity, functioning, as well as provided treatment and treatment outcomes. The proposed ICD-11 criteria have identified a fewer (or less frequently equal) number of cases with PTSD compared to ICD-10, DSM-IV, or DSM-5 in adult studies [9, 10, 14–17]. However, ICD-11- identified more cases with PTSD in one child sample when compared to DSM-IV and DSM-5 [18].

Diagnostic agreement between classification systems seems to vary. In one adult sample (*N =* 100), diagnostic agreement for PTSD with DSM-5 and the proposed ICD-11 criteria was found in 54 % of the cases [19]. In another sample of 510 injury patients followed 72 months after hospitalization, PTSD was diagnosed simultaneously in 64 % of the cases with both ICD-10 and the proposed ICD-11 criteria, and in 42 % of the cases with both the proposed ICD-11 and DSM-5 criteria among those meeting at least one PTSD criterion. The authors suggested that patients’ phenotypes may be different from one classification system to the other [16].

There are a no previous studies on how changes in PTSD criteria in the ICD-11 revision would impact upon diagnostics among youth. Further, assuming that the latent factor structure of PTSD is invariant across different age-groups and trauma types would be faulty. Therefore, we studied two samples of adolescents and young adults who had experienced similar types of mass shooting trauma. One-, two- and three factor solutions of the ICD-11 criteria similar to Forbes et al. [6] were evaluated here among the survivors of a less studied mass trauma type. Prevalence rates, diagnostic agreement and the clinical features of PTSD cases identified with ICD-11 were compared to the PTSD cases identified by ICD-10 and DSM-IV.

## Methods

### Participants and procedure

There have been two school shooting incidents in Finland during the last decade; at Jokela High School, 2007 and at Kauhajoki vocational school and college, 2008. We have studied the recovery process of the adolescents and young adults who were students of these institutions at the time of the incidents. The overall protocol for the study has been described elsewhere [20, 21].

Here we present the results from an interview arm of the study. Clinical psychiatric assessments were performed about 16 months after the index incident. Those who had consented to take part in the follow-up study were contacted by phone to schedule interviews. Subjects could refuse an interview but otherwise continue in the study. Ethical permissions were given and study protocols were accepted by the Ethics Committee of Helsinki University Central Hospital and the Ethics Committee of the Hospital District of South Ostrobothnia.

Of the 474 Jokela middle and high school students, 124 were interviewed, 26.2 % of all the students and 53.7 % of the students taking part in the baseline questionnaire (*N =* 231) four months after the incident. Of the 389 Kauhajoki educational institution students, 104 were interviewed, 26.7 % of all the students and 44.1 % of the students taking part in the baseline questionnaire (*N =* 236). There were 228 interviewed students in total, of which 32 (14.0 %) were interviewed by phone due to inconveniently long distances, the remainder being face-to-face interviews. There were 184 (80.7 %) females and 44 males (19.3 %) and the mean age was 17.6 years (SD = 3.7, median age 17, range 12–30 years). Two thirds of the interviewed sample had no previous trauma exposure (65.0 %), while one quarter (24.3 %) disclosed one previous traumatic event, and about one in ten (10.7 %) had experienced two or more previous traumatic experiences. New trauma had been experienced by 19 students (8.5 %) after the index trauma.

Those who took part in the interview arm of the study did not differ from the whole study sample on age, socioeconomic status, previous need for psychosocial support, previous trauma exposure, exposure level of the index trauma, or baseline levels of symptoms measured by self-report scales. Boys did not take part in the interview as often as girls in Jokela, *p =* 0.001. Those who had experienced a new trauma after the index event took part in the interview more often in Jokela, *p <* 0.001.

### Measures

The interview included basic background information. Exposure to the school shooting was used as the index incident. The students were asked to tell about their experience of the event in their own words, while structured questions were asked about fearing for their own or others lives or physical injury, about feelings of not being able to stop the events happening, requiring help, or acting in panic or being overwhelmed. The PTSD A2 criterion (exposure to a traumatic event is accompanied by intense fear, helplessness, or horror) required in DSM-IV was categorized based on these answers as either present (=1) or not present (= 0). An assessment of the severity of the exposure was based on the level of threat-to-life and losses suffered, as reported in the baseline questionnaire. The answers were categorized into mild-to-moderate, significant, and severe-to-extreme exposure [20].

The semi-structured Schedule for Affective Disorders and Schizophrenia for School-Age Children—Present and Lifetime version (K-SADS-PL) [22] was used with those under 18 years to assess major psychiatric diagnoses according to DSM-IV. The K-SADS-PL has from good to excellent test–retest reliability, high concurrent validity and inter-rater agreement for the original and translated versions [22–25]; the Finnish translation has previously been used in different kinds of study settings. Adult age students were interviewed using the Structured Clinical Interview for DSM-IV (SCID-I) to assess major psychiatric diagnoses [26]. However, the K-SADS-PL was used with all age groups for PTSD symptoms to ensure item by item consistency within the data. Psychosocial functioning was approximated using the Global Assessment Scale (GAS), using the children’s version when appropriate [27].

The interviewers were either adolescent psychiatrists or experienced psychiatric nurses trained to use the instrument. Scorings by the nurse interviewers were reviewed with psychiatrists. Ambiguities were settled by consensus between two psychiatrists.

Although the used K-SADS-PL interview is based on DSM-IV, it includes items that are the same or close approximates of the proposed six ICD-11 symptoms. The re-experiencing items were especially considered, since the ICD-11 criteria of nightmares and flashbacks require that the event is experienced as occurring again and typically with overwhelming emotions. This excludes, for example, the use of the K-SADS-PL item *Recurrent thoughts or images of event* for the ICD-11 diagnosis, since the question allows for a voluntary contemplation and not necessarily a re-experience of the trauma with strong emotions.

The interviewed items corresponding to the ICD-11 Beta Draft [3] PTSD diagnostic criteria for re-experiencing symptoms are *nightmares* (Probes: *Has there ever been a time when you had a lot of nightmares? … How did you feel when you woke up from one of your nightmares*?) and *dissociative flashbacks* (Probes: *Has there ever been a time when you felt like it was happening again? … Was the feeling so strong that it was hard to tell whether or not it was happening again? Have you ever seen or heard things that you knew weren’t really there that reminded you of what happened?*). Items corresponding to the avoidance symptoms were *efforts to avoid thoughts or feelings associated with the trauma* (Probes: *What kind of things do you do or have you done to keep from thinking about what happened? To get rid of bad thoughts, some kids, read, do things to keep busy, or go to sleep. Did you ever do any of these things or other things to get rid of those bad thoughts and/or feelings?*), and *efforts to avoid activities or situations that brought up recollections of the trauma* (Probes: *You said before that sometimes __ reminds you of what happened. Did you try to avoid __?*). Items corresponding to the hyperarousal symptoms were *hypervigilance* (Probes: *Since __ happened, are you more careful? Do you feel like you always have to watch what’s going on around you? Do you double check the doors or windows to make sure they are locked?*”), and *exaggerated startle response* (Probes: *Since __happened, are you more jumpy? Do little noises really scare you?*). Scorings of the PTSD symptom items in the K-SADS-PL are: 0 = *no information*, 1 = *the symptom is not present*, 2 = *the symptom is present*, i.e. the symptom criterion is fulfilled. Rare missing items were replaced by 0 = *no information*.

Scorings of the separate symptom and impairment items were used to compose PTSD diagnoses according to DSM-IV, ICD-10, and the proposed ICD-11 two- and three-factor solutions criteria. DSM-IV diagnosis required a fulfilling of stressor criterion A1 and A2 as well as1/5 of re-experiencing or intrusive symptoms, 3/7 avoidance symptoms, 2/5 hyperarousal symptoms, and impairment. The ICD-10 diagnosis was made when stressor criterion A1 was present as well as 1/4 re-experiencing symptoms, 1/2 avoidance symptoms, and specific amnesia or 2/5 hyperarousal symptoms. ICD-11 three-factor diagnosis required stressor criterion A1 as well as 1/2 of re-experiencing symptoms, 1/2 avoidance symptoms, 1/2 hyperarousal symptoms, and impairment. In comparison, the ICD-11 two-factor model combines re-experiencing symptoms and avoidance symptoms when 2/4 of these symptoms are required for the diagnosis (Table 1).

**Table 1:** Proportions of the studied subjects meeting PTSD symptom criteria and diagnoses

	DSM-IV	ICD-10	Three-factor ICD-11	Two-factor ICD-11
	*n* (%)	*n* (%)	*n* (%)	*n* (%)
Stressor criterion A				
A1. Traumatic event	228 (100.0)	228 (100.0)	228 (100.0)	228 (100.0)
A2. Emotional response	186 (81.6)			
Re-experiencing criterion B				
B1. Distressing recollections	133 (58.3)	133 (58.3)		
B2. Distressing dreams	117 (51.3)	117 (51.3)	117 (51.3)	117 (51.3)
B3. Sense or reliving, illusions, hallucinations, or dissociative flashbacks	52 (22.8)			
(B3.) Dissociative flashbacks only		44 (19.3)	44 (19.3)	44 (19.3)
B4. Psychological reactivity	89 (39.0)	89 (39.0)		
B5. Physiological reactivity	62 (27.2)			
Avoidance criterion C				
C1. Avoiding internal reminders	90 (39.5)	90 (39.5)	90 (39.5)	90 (39.5)
C2. Avoiding external reminders	55 (24.1)	55 (24.1)	55 (24.1)	55 (24.1)
C3. Specific amnesia	39 (17.1)	39 (17.1)		
C4. Diminished interest	48 (21.1)			
C5. Detachment	26 (11.4)			
C6. Restricted affect	45 (19.7)			
C7. Foreshortened future	17 (7.5)			
Hyperarousal criterion D				
D1. Difficulty sleeping	107 (46.9)	107 (46.9)		
D2. Irritability	64 (28.1)	64 (28.1)		
D3. Difficulty concentrating	96 (42.1)	96 (42.1)		
D4. Hypervigilance	88 (38.6)	88 (38.6)	88 (38.6)	88 (38.6)
D5. Exaggerated startle response	106 (46.5)	106 (46.5)	106 (46.5)	106 (46.5)
Criterion fulfilled				
Exposure and symptom criteria positive	52 (22.8)	**85 (37.3)**	66 (28.9)	74 (32.5)
Exposure and symptom criteria positive with positive impairment criteria	**43 (18.9)**	62 (27.2)	**51 (22.4)**	**56 (24.6)**

DSM-IV diagnosis requires stressor criterion A1 and A2 as well as 1/5 of re-experiencing symptoms, 3/7 avoidance symptoms, 2/5 hyperarousal symptoms, and impairment. ICD-10 diagnosis requires stressor criterion A1 as well as 1/4 re-experiencing symptoms, 1/2 avoidance symptoms, and specific amnesia OR 2/5 hyperarousal symptoms. ICD-11 three-factor diagnosis requires stressor criterion A1 as well as 1/2 of re-experiencing symptoms, 1/2 avoidance symptoms, 1/2 hyperarousal symptoms, and impairment. ICD-11 two-factor diagnosis requires stressor criterion A1 as well as 2/4 of re-experiencing symptoms or avoidance symptoms, 1/2 hyperarousal symptoms, and impairment

Bolded numbers indicate when the diagnostic criteria are met

PTSD diagnoses occurring after the index incident until the time of the interview were included in the analyses. Other psychiatric disorders present after the index incident were considered when studying comorbidity. Depression included a major depressive disorder single or recurrent episode, dysthymic disorder and depressive disorders NOS. Anxiety disorders included a general anxiety disorder, panic disorder with or without agoraphobia, agoraphobia, specific phobia, social phobia and anxiety disorder NOS. Rates of alcohol use disorders were low in this partly adolescent sample. We categorized alcohol use as a *no use or non-problem* use and *problem use. Problem use* was coded when at least one alcohol use disorder diagnostic criterion was met or when an adolescent engaged in heavy binge drinking.

Posttraumatic stress symptom severity was estimated with Impact of Event Scale version that has 22 items (IES-22) [21, 28]. This self-report form includes symptom statements (e.g. *I stayed away from reminders of it*) that are rated on the basis of how frequently they occurred during the past seven days; 0 = *not at all*, 1 = *rarely*, 3 = *sometimes*, 5 = *often*. The General Health Questionnaire 12-item version (GHQ-12) was used to evaluate general psychological symptoms [29]. The symptoms enquired (e.g. *Over the past few weeks, have you been feeling unhappy or depressed?* 0 = *not at all*, 1 = *no more than usual*, 2 = *rather more than usual*, 3 = *much more than usual*) are scored in a bimodal fashion (0–0–1–1). Sum scores for the scales were calculated and used as continuous variables. Missing items were replaced by the the respondent’s mean score of the other items on a given scale. The entire scale was omitted from the analyses when more than 15 % of the items were missing. Internal consistencies (Cronbach’s α) were 0.941 for IES-22 and 0.897 for GHQ-12 with this sample.

### Statistical analyses

The distributions of variables were presented as percentages for categorical variables and means (M) and standard deviations (SD) for continuous variables.

Confirmatory factor analysis (CFA) was used to compare three- and two-factor models for the six dichotomous K-SADS-PL symptom variables corresponding to the proposed ICD-11 PTSD criteria. The factors (and their indicators) in the three-factor model were 1) re-experiencing (distressing dreams, dissociative flashbacks), 2) avoidance (avoiding internal reminders, avoiding external reminders), and 3) hyperarousal (hypervigilance, exaggerated startle response). In the two-factor model, the four items from the re-experiencing and avoidance factors were combined to form one factor, while the hyperarousal factor remained intact. Models were analysed using the Weighted Least Squares Mean and Variance adjusted (WLSMV) estimator. Model fit was assessed using the *χ*^2^ statistic, the Root Mean Squared Error of Approximation (RMSEA), the Comparative Fit Index (CFI), and the Tucker–Lewis Index (TLI). A RMSEA below 0.06 and a CFI/TLI above 0.95 was considered to indicate a good fit [30]. Additionally, Bayesian Information Criteria (BIC) were obtained from models estimated with a maximum likelihood (ML) estimator, where lower BIC values suggest a better fit.

Comparisons between proportions of the PTSD cases (prevalence rates) diagnosed by the different criteria were made with a Z-score test, while differences in background information and clinical characteristics among youth having a particular PTSD diagnosis, compared to those not having the diagnosis, were tested using an analysis of variance (ANOVA) and chi-square test. Cohen’s kappa was calculated to measure the diagnostic agreement between the proposed three-factor ICD-11 PTSD caseness and the other diagnostic systems.

Those individuals who met the three-factor ICD-11 PTSD criteria also met the two-factor ICD-11 criteria, and the ICD-10 criteria with and without impairment, the first one having the strictest criteria. Differences between clinical characteristics were tested between those meeting both diagnostic criteria and those meeting the less strict criteria only. Comparison between the three-factor ICD-11 and DSM-IV PTSD criteria was made between groups of those meeting both diagnoses, and those meeting the three-factor ICD-11 or DSM-IV diagnoses only. ANOVAs, with post hoc multiple comparisons (Bonferroni) when appropriate, and chi-square tests were used.

A significance level of *p <* 0.05, two-tailed, was chosen. Analyses were performed using IBM SPSS Statistics version 22 and Mplus 7.1 software [31].

## Results

### Testing the ICD-11 factor models

Using the WLSMV estimator, the correlation between the latent factors of re-experiencing and avoidance was estimated to have a value above 1.0 in the three-factor CFA model. This indicates problems in model specification, i.e. that the two factors are not statistically distinguishable, and suggests that these two factors should be combined. Also a model estimated using the ML estimator produced a very high correlation (r = 0.99) between these two factors. A model comparison using BIC values from the ML estimation indicated that the two-factor model had a better fit (1499.134) than the three-factor model (1509.479). The two-factor model showed a good fit to the data (*χ*^2^ [8] = 5.31, *p =* 0.724; CFI > 0.99; TLI = 1.01; RMSEA (90 % CI) < 0.01 (0.00-0.06) using the WLSMV estimator and also a significantly better fit compared to the one-factor solution (WLSMW *χ*^2^-difference [1] = 5.42, *p =* 0.020).

The factor loadings in the two-factor model (WLSMV) were as follows: 1) for the re-experiencing/avoidance factor: distressing dreams 0.58, dissociative flashbacks 0.77, avoiding internal reminders 0.74, and avoiding external reminders 0.87; and 2) for the hyperarousal factor: hypervigilance 0.78 and exaggerated startle response 1.00. Correlation between the two latent factors was r = 0.86.

### Prevalence rates of PTSD symptoms

The proportions of the subjects meeting the different PTSD symptom criteria are shown in Table 1. The most often disclosed ICD-11 PTSD symptom was nightmares, while flashbacks were the least often reported symptoms. Pertaining to the DSM-IV re-experiencing items, although *Recurrent thoughts or images of event* was reported very often (58.3 %), it was not used for the proposed ICD-11 diagnosis here, since the question includes voluntary contemplation and not necessarily re-experiencing the trauma in the present accompanied by strong and overwhelming emotions.

### Prevalence rates of PTSD according to the different classification systems

The prevalence rate of PTSD with DSM-IV was 18.9 %, with ICD-10 the highest at 37.3 %, and with the proposed three-factor ICD-11 criteria at 22.4 % (Table 2). When the two-factor solution was used with the proposed ICD-11 criteria, the prevalence rate of PTSD was 24.6 %, which was not statistically different from the three-factor model (Z-score = 0.553, *p =* 0.582). Differences between proportions of PTSD cases were not significant with DSM-IV and the proposed 2- or 3-factor ICD-11 classifications. The proportion of PTSD cases diagnosed with ICD-10 was significantly higher than all the other classifications systems (e.g. 3-factor ICD-11: Z-score = 3.480, *p <* 0.001; DSM-IV: Z-score = 4.377, *p <* 0.001). If impairment was added to the ICD-10 PTSD criteria, then the proportion of the ICD-10 cases compared to ICD-11 was no longer significant, albeit the difference compared to DSM-IV remained (Z-score = 2.305, *p =* 0.021).

**Table 2:** Diagnostic agreement of the proposed three-factor ICD-11 PTSD criteria with DSM-IV, ICD-10 and two-factor ICD-11 PTSD criteria

PTSD status	Three-factor ICD-11		
	yes, *n =* 51 (22.4 %)	no, *n =* 177	
	*n* (%, from the whole sample)		Kappa
DSM-IV			.679***
yes, *n =* 43 (18.9 %)	35 (15.4)	8 (3.5)	
no, *n =* 185	16 (7.0)	169 (74.1)	
ICD-10			.653***
yes, *n =* 85 (37.5 %)	51 (22.4)	34 (14.9)	
no, *n =* 143	0 (0.0)	143 (62.7)	
ICD-10 with impairment			.871***
yes, *n =* 62 (27.2 %)	51 (22.4)	11 (4.8)	
no, *n =* 166	0 (0.0)	166 (72.8)	
Two-factor ICD-11			.939***
yes, *n =* 56 (24.6 %)	51 (22.4)	5 (2.2)	
no, *n =* 172	0 (0.0)	172 (75.4)	

***p < 0.001

### Diagnostic agreement

Table 2 shows the diagnostic agreement between the three-factor solution ICD-11 criteria and the other classification systems. Two- and three-factor ICD-11 solutions were in excellent diagnostic agreement, kappa > 0.9. Meanwhile ICD-10 and DSM-IV were only at a moderate level of diagnostic agreement with the three-factor ICD-11. ICD-10 identified a large number of PTSD cases (*n =* 34, 14.9 % of the whole sample) to be present when the three-factor ICD-11 diagnostic criteria were not fulfilled. If the ICD-10 diagnostic criteria were complimented with the impairment criterion, the diagnostic agreement changed to be good with ICD-11 (kappa = 0.87).

### Clinical characteristics of the PTSD cases identified by the different classification systems

All the diagnostic systems found PTSD status to be associated with female sex, a more severe level of exposure, lower levels of psychosocial functioning and higher levels of posttraumatic and general psychological symptoms (Table 3). Depression comorbidity was also associated with the PTSD status in all the diagnostic groups, while anxiety disorder comorbidity or alcohol problem use were not associated with the PTSD status. ICD-10 identified a larger proportion of PTSD cases compared to the three-factor ICD-11 model among females (Z-score = 3.296, *p <* 0.001), among both studied groups (Jokela: Z-score = 2.107, *p =* 0.035; Kauhajoki: Z-score = 2.842, *p =* 0.005) and among lower level of exposure groups (mild-to-moderate: Z-score = 2.068, *p =* 0.038; significant: Z-score = 2.903, *p =* 0.004; severe-to-extreme Z-score = 1.212, *p =* 0.226). Duration of symptoms was assessed while keeping DSM-IV criteria in mind during the interview. PTSD was chronic (not remitted by 16 months) in one third of the cases (*n =* 14, 34.1 %), while duration of remitted PTSD had been over four months on average (M = 134 days, SD = 101 days) among those cases assessed with the DSM-IV criteria.

**Table 3:** Demographics and clinical characteristics of the interviewed sample and the PTSD cases identified by DSM-IV, ICD-10 and the proposed ICD-11 two- and three factor models

		PTSD cases by diagnostic system							
	WHOLE SAMPLE	DSM-IV *n =* 43 (18.9 %)		ICD-10 *n =* 85 (37.3 %)		Two-factor ICD-11 *n =* 56 (24.6 %)		Three-factor ICD-11 *n =* 51 (22.4 %)	
	*N =* 228		*χ* ^2^/F^a^		*χ* ^2^/F^a^		*χ* ^2^/F^a^		*χ* ^2^/F^a^
Sex, *n* (%)			7.30**		10.65**		7.04**		7.59**
Male	44 (19.3)	2 (4.7)		7 (8.2)		4 (7.1)		3 (5.9)	
Female	184 (80.7)	41 (95.3)		78 (91.8)		52 (92.9)		48 (94.1)	
Age, M (SD)	17.6 (3.7)	17.2 (3.8)	.48	17.6 (3.9)	.04	17.6 (3.8)	.00	17.3 (3.8)	.35
Range 12–30 years									
Study group, *n* (%)			.02		.79		.58		.01
Jokela	124 (54.4)	23 (53.5)		43 (50.6)		28 (50.0)		28 (54.9)	
Kauhajoki	104 (45.6)	20 (46.5)		42 (49.4)		28 (50.0)		23 (45.1)	
Exposure, *n* (%)			20.40***		16.51**		19.89**		20.93***
Mild-to-moderate	55 (24.1)	5 (11.6)		10 (11.8)		4 (7.1)		3 (5.9)	
Significant	124 (54.4)	18 (41.9)		48 (56.5)		30 (53.6)		27 (52.9)	
Severe-to-extreme	49 (21.5)	20 (46.5)		27 (31.8)		22 (39.3)		21 (41.2)	
GAS, M (SD)	75.9 (11.1)	64.1 (11.6)	78.96***	69.1 (11.8)	65.18***	67.2 (12.0)	53.86***	66.8 (11.8)	51.84***
GHQ-12, M (SD)^b^	3.3 (3.3)	6.5 (3.5)	62.39***	4.7 (3.6)	29.00***	6.1 (3.4)	66.64***	5.9 (3.5)	46.35***
IES-22, M (SD)^b^	29.2 (23.7)	53.4 (25.0)	68.60***	44.8 (24.9)	73.37***	53.1 (22.2)	104.83***	53.0 (23.0)	85.04***
Depression, *n* (%)	20 (8.8)	8 (18.6)	6.40*	15 (17.6)	13.34***	11 (19.6)	10.96**	10 (19.6)	9.64**
Anxiety disorder, *n* (%)	23 (10.1)	7 (16.3)	2.24	12 (14.1)	2.43	7 (12.5)	.48	6 (11.8)	.20
Alcohol problem use, *n* (%)	29 (12.8)	6 (14.0)	.07	10 (11.8)	.13	8 (14.5)	.20	8 (15.7)	.50

^a^PTSD vs. no PTSD within the diagnostic classification system

^b^reported four months after the incident

*p < 0.05, **p < 0.01, ***p < 0.001

#### Cases identified by both the three-factor and two-factor ICD-11 vs. two-factor ICD-11 only

There were only five extra PTSD cases that the two-factor ICD-11 diagnostic criteria identified over the original three-factor model. These five cases did not differ significantly on any tested characteristics from those diagnosed by both models. The tested characteristics were age, sex, exposure severity, psychosocial functioning GAS scores, general psychological symptoms with GHQ-12, posttraumatic stress symptoms with IES-22, and comorbidity with depression, anxiety disorder and alcohol problem use.

#### Cases identified by both the three-factor ICD-11 and ICD-10 vs. ICD-10 only

A total of 34 PTSD cases were identified using the ICD-10 diagnostic criteria that were not identified using the three-factor ICD-11. Although those cases did not differ from the cases diagnosed by both diagnostics systems on age, sex or comorbidity, they did have better psychosocial functioning, (F [1,81] = 4.499, *p =* 0.037), less severe symptoms (GHQ-12: F [1,79] = 13.796, *p <* 0.001; IES-22: F [1,79] = 14.828, *p <* 0.001) and they had experienced less severe exposure (*χ*^2^ [2] = 7.587, *p =* 0.023). When an impairment criterion was imposed on ICD-10, there were 11 cases remaining that were not simultaneously diagnosed with the ICD-11 criteria. The differences in psychosocial functioning and general psychological symptoms between the diagnostic groups were no longer significant. However, these 11 cases had less severe posttraumatic stress symptoms scores (IES-22: M = 53.0 vs. M = 29.3, F [1,56] = 8.837, *p =* 0.004) and they had experienced less severe exposure (Fisher’s exact = 8.547, *p =* 0.010) compared to the cases identified with both ICD-10 and three-factor ICD-11.

#### Cases identified by both the three-factor ICD-11 and DSM-IV vs. ICD-11 or DSM-IV only

There was some discrepancy between the ICD-11 and DSM-IV diagnostic criteria. Both criteria mutually identified 35 PTSD cases, while the three-factor ICD-11 identified eight cases and the DSM-IV 16 cases that did not meet the other criteria. There were no differences between these groups on age, sex, symptom severity or comorbidity. Those cases only meeting the ICD-11 criteria had higher levels of psychosocial functioning than cases meeting both criteria, although there was no significant difference to those only identified by the DSM-IV (F [2,54] = 3.415, *p =* 0.040; Bonferroni post hoc tests both vs. ICD-11 only *p =* 0.043; both vs. DSM-IV only *p =* 1.000; ICD-11 vs. DSM-IV *p =* 0.223). Cases identified by both criteria were more often in the severe-to-extreme exposure group, while the cases only fulfilling the DSM-IV criteria were more often mild-to-moderately exposed compared to the cases only fulfilling the ICD-11 criteria (both diagnoses: mild-to-moderate exposure 5.7 %, significant 40.0 %, severe-to-extreme 54.3 %; ICD-11 only: mild-to-moderate 6.3 %, significant 81.3 %, severe-to-extreme 12.5 %; DSM-IV only: mild-to-moderate 37.5 %, significant 50.0 %, severe-to-extreme 12.5 %; Fisher’s exact 14.656, *p =* 0.002).

## Discussion

The way that psychiatric disorders related to traumatic stress are categorized has an impact on who is treated for posttraumatic symptoms and how neurobiological and psychological phenomena related to different disorders are studied.

We studied the three-factor ICD-11 model of PTSD symptoms using confirmatory factor analysis. Our results indicated that the re-experiencing and avoidance factors of this proposed model are not statistically distinguishable, likely representing a single factor. Further, model comparisons using BIC values suggested that the two-factor model, where these two factors are combined, has a better fit to the data over the three-factor model. The fit indices for the two-factor model were excellent; there were only a few extra cases identified with the two-factor model compared to the three-factor model and these cases did not differ in background or clinical features from cases identified with both the three—and two-factor models. Consequently, our data suggest that the two-factor model is preferable to the three-factor model. A previous study by Forbes et al. [6] suggested that the two-factor model is preferable as it has at least an equivalent fit with the three-factor model. Other studies have not scrutinized the latent factor structure of the ICD-11 PTSD but have managed to show that the three-factor model has a good model fit [9, 10].

The proposed ICD-11 criteria are more restrictive compared to the ICD-10 criteria. It was found that the ICD-10 criteria identify a significantly larger proportion of PTSD cases with better psychosocial functioning and less severe symptoms. However, the ICD-10 has been criticized for lacking the requirement for impairment in functioning. When this criterion was imposed on the ICD-10, it could no longer identify a significantly larger proportion of PTSD cases compared to the ICD-11. This difference seems to explain the majority of the divergence between the 10th and 11th ICD versions. However, some differences in clinical features remained, as remaining cases with only the ICD-10 diagnosis had less severe posttraumatic stress symptoms and had experienced less severe exposure.

Diagnostic agreement between the DSM-IV and ICD-11 criteria was moderate. ICD-11 identifies more PTSD cases than DSM-IV (*n =* 51 vs. *n =* 43) although the difference was not statistically significant. The youth meeting the ICD-11 criteria only had higher levels of psychosocial functioning than cases meeting both criteria. Those meeting only the DSM-IV criteria were less severely exposed to trauma compared to those only meeting the ICD-11 criteria.

Studies with adult samples have identified mainly lower prevalence rates of PTSD when assessed with the ICD-11 criteria compared to the ICD-10 and DSM-IV criteria [9, 14–17]. Our results are somewhat contrary, as the ICD-11 criteria identified more cases than the DSM-IV criteria and slightly fewer cases than the ICD-10 criteria with impairment, albeit neither were statistically significant. One preliminary study with children indicated that the ICD-11 identified more cases with PTSD compared to the DSM-IV and DSM-5 [18]. More studies are needed with child and adolescent samples to discover the kind of role that age and developmental stage have on the diagnostic thresholds of these different criteria.

In conclusion, the different diagnostic PTSD classifications are in a moderate to good agreement, despite identifying individuals with somewhat divergent clinical features.

### Limitations

The study was carried out before publication of DSM-5 and some of the new PTSD symptom criteria for negative alterations in mood and cognition could not be assessed. Thus, comparisons with DSM-5 PTSD could not be made.

Caution should be exercised when comparing the characteristics of the PTSD cases identified by the different diagnostic classifications as the sizes of the compared groups were too small in some instances to detect differences: for example there were only five extra cases using the two-factor ICD-11 criteria to compare with the 51 cases fulfilling both the two- and three-factor criteria.

This study used a sample of individuals with a similar type of index trauma. It could be argued that this does not capture the variety of PTSD among young people. However, this reduced variability in exposure types could also be seen as a strength when performing confirmatory factor analyses and when exploring the differences between the diagnostic systems.

PTSD symptom items assessed with the K-SADS-PL interview are dichotomous, which might warrant for cautiousness when comparing our results to the studies using measures with Likert-type or ordinal response scales. However, our results regarding factor structure and factor loading of the PTSD symptom items resemble well those reported by Forbes et al. [6] using five-point ordinal scale and the same WLSMV estimation method allowing categorical or ordinal factor items.

## Conclusions

The findings in this study indicate that the two-factor model of ICD-11 PTSD is preferable to the three-factor model. ICD-10 PTSD criteria were found to be lax compared to the proposed ICD-11 criteria. Interestingly, ICD-11 identified more PTSD cases than DSM-IV, which is in contrast to most previous studies with adult samples. Divergence of these diagnostic systems warrants further study across different ages, developmental levels and trauma types.

### Ethics approval and consent to participate

Ethical permissions were given and study protocols accepted by the Ethics Committee of Helsinki University Central Hospital and the Ethics Committee of the Hospital District of South Ostrobothnia. Participation was voluntary and each participant was asked to sign an informed consent form. Signed informed consent was required from a parent or guardian of the students under 15 years according to Finnish legislation. Parents of minors were informed and they had the opportunity to deny participation.

### Availability of data and materials

Data is available and may be requested from the corresponding author.

## Abbreviations

ANOVA: analysis of variance
BIC: Bayesian information criteria
CFA: confirmatory factor analysis
CFI: comparative fit index
CI: confidence interval
DSM: Diagnostic and Statistical Manual of Mental Disorders
GAS: Global Assessment Scale
GHQ: General Health Questionnaire
ICD: International Classification of Diseases
IES: Impact of Event Scale
K-SADS-PL: Schedule for Affective Disorders and Schizophrenia for School-Age Children—Present and Lifetime version
ML: maximum likelihood
PTSD: posttraumatic stress disorder
RMSEA: root mean squared error of approximation
TLI: Tucker–Lewis index
WHO: World Health Organization
WLSMV: weighted least squares mean and variance adjusted
